# Extended influenza seasons in Australia and New Zealand in 2025 due to the emergence of influenza A(H3N2) subclade K viruses

**DOI:** 10.2807/1560-7917.ES.2025.30.49.2500894

**Published:** 2025-12-11

**Authors:** Clyde Dapat, Heidi Peck, Lauren Jelley, Tanya Diefenbach-Elstob, Tegan Slater, Saira Hussain, Phillip Britton, Allen C. Cheng, Tim Wood, Annaleise Howard-Jones, Yi Mo Deng, Jessica E. Miller, Q. Sue Huang, Ian G. Barr

**Affiliations:** 1 WHO Collaborating Centre for Reference and Research on Influenza, VIDRL, Doherty Institute, Melbourne, Australia; 2 New Zealand Institute for Public Health and Forensic Science, Wallaceville, Upper Hutt, New Zealand; 3 Department of Infectious Diseases and Microbiology, The Children’s Hospital at Westmead, Westmead, Australia; 4 Sydney Infectious Diseases Institute, University of Sydney, Sydney, Australia; 5 Monash Infectious Diseases Service, Monash Health and School of Clinical Sciences, Monash University, Clayton, Australia; 6 Department of Microbiology and Immunology, University of Melbourne, Melbourne, Australia; * These authors contributed equally to this work and share first authorship.

**Keywords:** H3N2, A-subtype, subclade, epidemic, seasonal influenza, Oceania

## Abstract

In Australia and New Zealand, late outbreaks of an A(H3N2) variant virus termed subclade K extended the 2025 influenza season. Subclade K viruses were genetically and antigenically distinct from the 2025 vaccine A(H3N2) strain A/Croatia/10136RV/2023 (H3N2)-like virus and previously circulating subclade J viruses. Subclade K viruses have since been detected in over 34 countries and appear to have spread globally, except in South America. It is thus likely that they will further expand during the northern hemisphere winter 2025/26 season.

In 2025, influenza seasons in Australia and New Zealand were each prolonged due to the emergence of an influenza A(H3N2) variant of subclade K (formerly J.2.4.1). We describe the influenza epidemics overall in each country, with the phylogenetic characterisation of circulating viruses, and assess the dissemination of subclade K viruses, which thereafter were identified in most parts of the world. Antigenic characterisation of subclade K viruses found these to be distinct from prior circulating subclade J viruses and from the A(H3N2) strain A/Croatia/10136RV/2023 (H3N2)-like virus, which was included in the 2025 southern hemisphere (SH) and 2025/26 northern hemisphere (NH) vaccines. 

## Seasonal 2025 influenza epidemics in Australia and New Zealand

**Figure 1:**
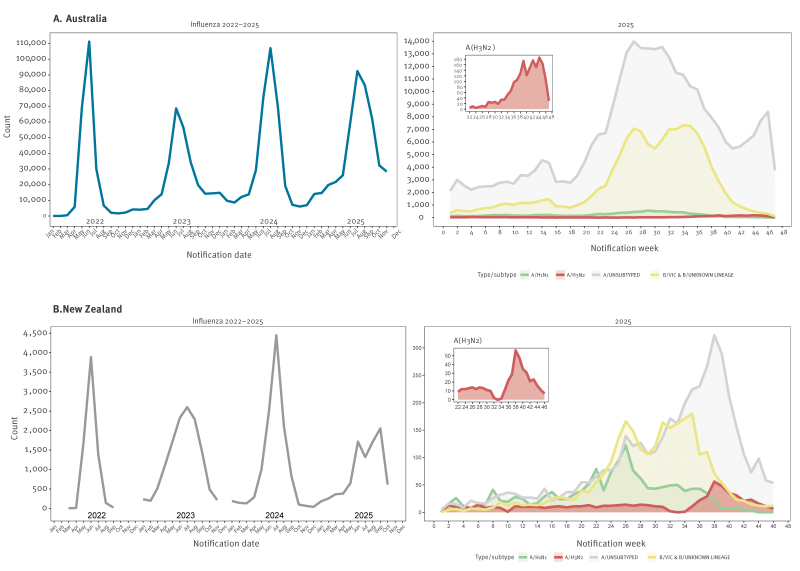
Numbers of laboratory-confirmed influenza cases by month of notification in 2022−2025 and numbers of cases stratified by type and subtype, by week of notification in 2025 for (A) Australia and (B) New Zealand, respectively

ISO: International Organization for Standardization; NNDSS: National Notifiable Diseases Surveillance System.

Influenza seasons vary somewhat each year in their onset, intensity, severity and duration. Many factors contribute to this variability such as temperature, rainfall, humidity, circulating virus types/subtypes, population immunity (vaccination or natural infections), as well as domestic and international travel [1-3]. The 2025 Australian influenza season had record numbers of laboratory-confirmed influenza cases since influenza became a notifiable disease in 2001 (457,906 cases from 1 January to 28 November 2025) [4] and an unusually long season stretching from May to November (Figure 1A). New Zealand had a more moderate season but with a longer than usual tail (Figure 1B) [5].

A. Left panel: Australian NNDSS laboratory-confirmed influenza cases for 2022−2025; right panel: Australian NNDSS laboratory-confirmed cases by type and subtype for 2025 with the inset showing A(H3N2) cases from ISO weeks 22−46.

B. Left panel: New Zealand laboratory confirmed cases for 2022−2025; right panel: New Zealand laboratory-confirmed cases by type and subtype for 2025 with the inset showing A(H3N2) cases from ISO weeks 22−46.

Note that only a small proportion of influenza A viruses are subtyped by Australian laboratories reporting to NNDSS (A. right panel), while somewhat more subtyping is performed in New Zealand (B. left panel).

In both countries the dominant influenza type/subtype across the 2025 season was influenza A(H1N1)pdm09 with influenza B co-circulating at lower levels [5,6]. Following a relative paucity in the earlier part of the season, A(H3N2) cases began to increase notably in August 2025 and became predominant in September in New Zealand and October−November in Australia (Figure 1). Major changes in influenza types/subtypes or the emergence of a variant at the end of an influenza season are relatively uncommon (except for influenza B viruses [7]).

## Phylogenetic analyses of 2025 H3N2 viruses in Australia and New Zealand and assessment of subclade K viruses spread worldwide

**Figure 2:**
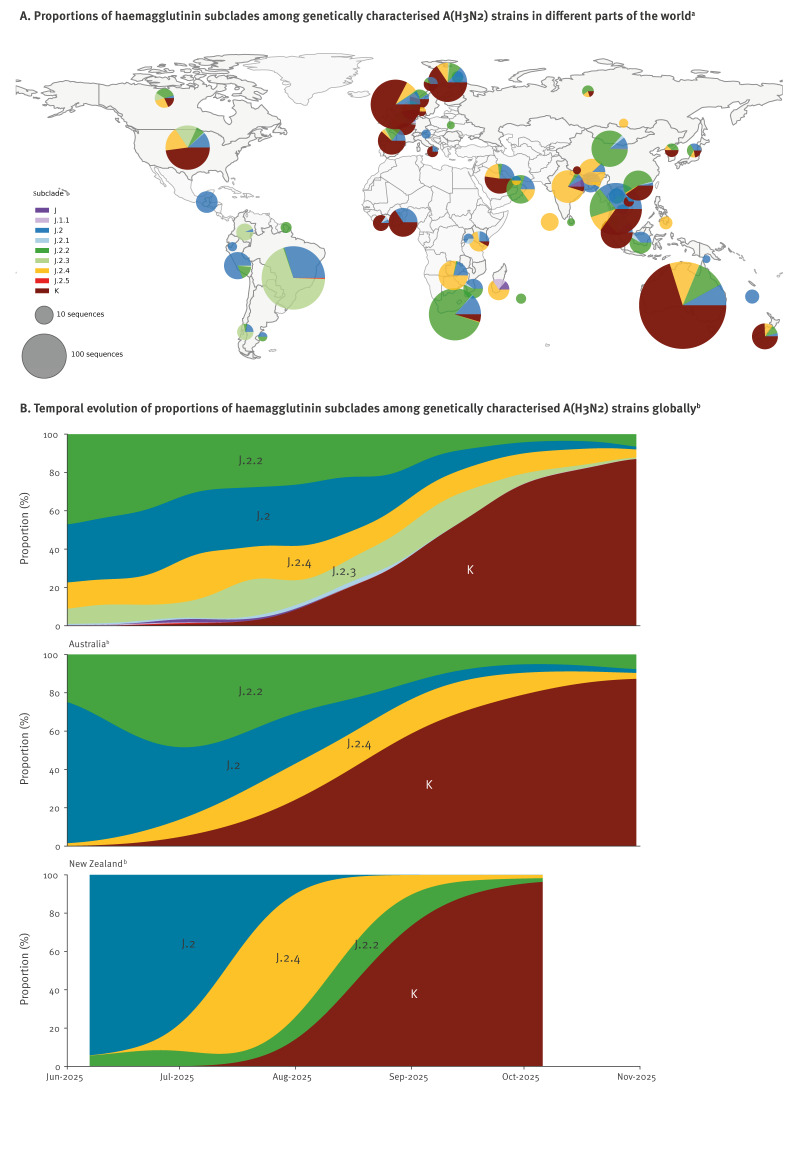
(A) Proportions^a^ of haemagglutinin subclades among genetically characterised A(H3N2) strains in different parts of the world, and (B) temporal evolution of these proportions globally, in Australia (June−November), and in New Zealand, (June−October 2025)^b^

Influenza A(H3N2) viruses received from 2025 from Australia (n = 1,419) and New Zealand (n = 52) were analysed by genetic sequencing and phylogenetic analysis (n = 998 and n = 49, respectively) and by antigenic analysis (n = 205 and n = 35, respectively). A phylogenetic analysis of the haemagglutinin (HA) gene of these viruses revealed a striking change over the season with respect to the frequencies of the various HA subclades. For the early part of the season the J.2 or J.2.2 subclade viruses predominated (Nextclade nomenclature [8,9]) with a small number of J.2.4 viruses, however, from August onwards a new subclade (initially called subclade J.2.4.1 now termed subclade K) emerged. First detected in Sydney and Melbourne on 17 July 2025 (A/Sydney/429/2025, A/Victoria/2282/2025), subclade K viruses spread rapidly throughout Australia, while in New Zealand the first subclade K virus detection was in Auckland on 27 August 2025 (Figure 1, Figure 2).

WHO: World Health Organization.

^a^ Pie area is proportional to the total number of sequences sampled per country. Plots are based on available haemagglutinin sequences from GISAID and may not accurately represent the situation in countries and do not take into account countries not sequencing viruses or not uploading sequences to GISAID.

^b^ Plots are based on haemagglutinin sequences available from WHO Influenza Centre in Melbourne and from GISAID.

As outlined in the Supplementary Material, the figures represent all available A(H3N2) sequences available on GISAID up to 11 November 2025, with collection dates from 1 June 2025 onwards.

Overall, from A(H3N2) viruses collected in 2025 and sequenced, the subclade K viruses constituted approximately half (502/998) of the Australian viruses and more than two thirds (35/49) of the New Zealand viruses. As shown in Supplementary Figure 1, compared with earlier J.2.4 viruses such as A/Sydney/1359/2024, subclade K viruses have several additional amino acid substitutions in their HA (K2N, N158D, I160K, T328A, Q173R, S378N and S144N which adds a potential N-glycosylation site). The K subclade virus neuraminidase (NA) genes also formed a distinct clade with a D346G amino acid change that separate them from J.2.4 NA genes (Supplementary Figure 1).

**Figure 3:**
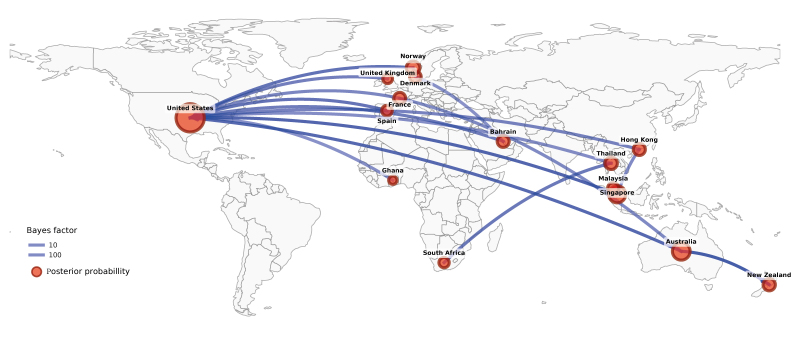
Global migration flow map of A(H3N2) subclade K viruses inferred from Bayesian phylogeographic analysis, 2025

A phylogeographic analysis showed that Australian/New Zealand A(H3N2) K viruses may have been imported/originated from the United States (US), as the first K virus sequence on GISAID [10] was from New York on 23 June 2025 (EPI_ISL_20126669); subsequently other detections occurred in Wisconsin and Michigan in July 2025, with similar timing to the first detections in Australia. This analysis also strongly suggested that Australia was the source of K viruses responsible for the New Zealand outbreaks (Figure 3). Globally GISAID data suggest these A(H3N2) subclade K viruses have now been detected in at least 34 countries across the world to date including the US, some countries in Europe (with 13 in Western Europe) as well as in Asia, Africa, and the Middle East (Figure 2), based on sequences lodged on GISAID at the end of November 2025.

Blue lines represent statistically supported migration events between countries with line thickness proportional to the Bayes factor. Red circles represent the posterior probability of migration events associated with each country. Data are based on available haemagglutinin sequences from GISAID. Details of the analysis are provided in the Supplementary Material.

## Antigenic characterisation of A(H3N2) viruses of various subclades including the K subclade

**Figure 4:**
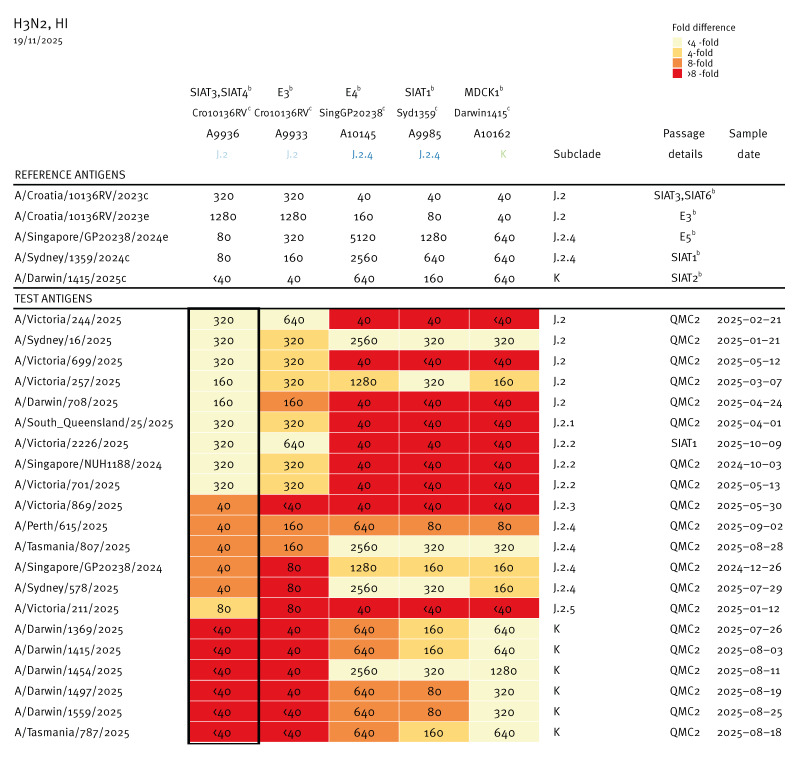
Haemagglutination inhibition titres of ferret post-infection antisera raised against various subclades of influenza A(H3N2) reacting with two isolates from 2024^a^ and 19 Australian virus isolates generated from samples with collection dates in 2025^a ^(n = 21 test isolates)

When Australian K subclade viruses were tested antigenically by haemagglutination inhibition (HI) assays using post-infection ferret antisera, the K subclade viruses were poorly inhibited by ferret antiserum to the 2025 SH vaccine cell-grown virus A/Croatia/10136RV/2023, a J.2 virus. Indeed, of 205 K subclade virus isolates tested, 204 (99.5%) showed ≥ 8-fold reactivity reductions to this antiserum compared to the homologous A/Croatia/10136RV/2023 titre. On the other hand, the isolates were well inhibited by antisera to J.2.4 viruses, A/Singapore/GP20238/2024 or A/Sydney/1359/2024, which are included in the 2026 SH vaccine, since among 205 isolates tested, only seven (3.4%) showed ≥ 8-fold reductions to the homologous A/Sydney titre. K subclade virus isolates were also well inhibited by an antiserum to a subclade K virus (A/Darwin/1415/2025) whereby only one of 137 isolates tested (0.7%) showed ≥ 8-fold reductions compared to the homologous A/Darwin titre (Figure 4). Similar results were obtained from New Zealand isolates, as presented in Supplementary Table 1.

c: cell grown virus; E: egg grown virus; HI: haemagglutination inhibition; MDCK: Madin−Darby canine kidney cell grown virus; QMC: qualified manufacturing cell grown virus; SIAT: SIAT-MDCK cell grown virus.

^a^ The two A(H3N2) virus isolates from 2024 tested for haemagglutination assays were of J.2.2. and J.2.4 subclades, and from Singapore. Australian test isolates were semi-randomly selected to cover different H3N2 subclades that circulated during 2025. Subclade K viruses were chosen based on their sampling on different days and locations (Darwin and Tasmania).

^b^ Passage details include whether the vaccine strains were cell (SIAT) or egg (E) grown. The SIAT and E indications are followed by a number, indicating the number of times strains were passaged.

^c^ Cro10136RV: A/Croatia/10136RV/2023; Darwin1415: A/Darwin/1415/2025; SingGP20238: A/Singapore/GP20238/2024; Syd1359: A/Sydney/1359/2024.

J.2, J.2.4 and K represent the A(H3N2) subclade nomenclature.

Each column represents a ferret antiserum raised to a particular virus and each row represents either a reference antigen virus (to which an antiserum was raised) or a test antigen virus representing various influenza virus isolates from Australia or Singapore. The test antigens are heat mapped according to the legend in the top right corner, with red representing > 8-fold reductions in titre compared with the homologous titre as in the same column (reference antigen section) in bold font.

Testing by HI assay against a cell-grown K virus (A/Darwin/1415/2025) was performed using Australian post-vaccination sera from 18−64-year-olds (n = 25) and older adults (≥ 65 years old; n = 20) who had received the 2025 southern hemisphere recommended vaccine (containing A(H3N2) A/Croatia/10136RV/2023-like viruses). Sera from people who were given an egg-based vaccine had geometric mean titre (GMT) reductions of 76% for 18−64-year-olds and 59% for older adults respectively, when compared with the GMT’s obtained to the cell-based vaccine virus A/District of Columbia/27/2023 (which is an A/Croatia/10136RV/2023-like virus). Sera from 18−64-year-olds who had been given a cell-based vaccine (n = 25) had a 44% reduction in the GMT compared with the titre of cell-based vaccine virus A/District of Columbia/27/2023. Smaller reductions were seen when these human post-vaccination sera were tested against subclade J.2 or J.2.2 virus isolates but large reductions were also seen with J.2.3 and J.2.5 subclade viruses, as depicted in the Supplementary Figure 2. The K viruses were therefore antigenically distinct from subclade J.2 and J.2.2 viruses that circulated widely in the earlier part of the 2025 season (Figure 2, Supplementary Figure 1).

## Demographic and disease severity findings

Influenza-positive samples were received from national general practitioner sentinel surveillance systems, hospital laboratories, private pathology laboratories and National Influenza Centres in Australia and New Zealand, and details can be found in the Supplementary Appendix. Individuals in Australia infected with influenza A(H3N2) viruses were considerably younger with median ages of 25 years for J.2/J.2.2 (interquartile range (IQR): 5−60 years; n = 368) and 20 years for K (IQR: 8−53 years; n = 502) than those with A(H1N1)pdm09 (median age: 37 years, IQR: 5−66 years; n = 5,219) but more similar in age to influenza B cases (median age: 18 years, IQR: 6−29 years; n = 2,086).

A comparison of the demographics of Australian individuals infected with the subclade A(H3N2) viruses J.2 and J.2.2 compared with K viruses in 2025 showed slight differences in their median ages. People infected with J.2 and J.2.2 subclade viruses had a median age of 25 years, while those infected with K subclade viruses had a median age of 20 years with age ranges from the 25^th^−75^th^ percentile of 5 years to 60 years (minimum and maximum ages: 0 years, 96 years) and 8 years to 53 years (minimum and maximum ages: 0 years, 94 years), respectively, as shown in the Supplementary Table 2.

An analysis of 2025 influenza A(H3N2) cases at a major paediatric hospital in the state of New South Wales showed that K viruses made up most their hospitalised influenza A(H3N2) cases in September−October (39/50), as described in Supplementary Table 3. Median age was higher in K subclade cases (5.3 years; IQR: 1.5−10.2 years; n = 39) compared with non-K cases (2.6 years; IQR: 1.5−6.4 years; n = 30). Intensive care unit admissions were low across all subclades with only one admission in a J.2 subclade case. There were no deaths at 30 days.

## Discussion

The rapid rise of A(H3N2) influenza cases at the end of long influenza seasons in both Australia and New Zealand, provides evidence that the new subclade K virus variant is virologically fit and antigenically distinct from previously circulating H3N2 viruses. Based on the antigenic changes in the HA of the K viruses, the 2025/26 H3N2 NH vaccine component (i.e. A/Croatia/10136RV/2023-like virus) may have reduced effectiveness if K viruses circulate widely, and could result in increased cases and hospitalisations compared with recent years when A(H1N1)pdm09 predominated in many regions across Europe, Asia and North America [7].

This is the first time that such a variant has emerged so rapidly and spread so widely towards the end of the season in Australia−New Zealand and has continued to circulate into summer in Australia. This is unusual for A(H3N2) viruses but has been seen previously on occasions in Australia with influenza B viruses [11]. Late emerging A(H3N2) viruses also occurred in 2019 that resulted in a delay in the recommendation for the 2019/20 NH influenza vaccine A(H3N2) component, with an A/Kansas/14/2017-like virus finally being selected [12], and earlier in 2003 when A/Fujian/411/2002-like viruses emerged late in the 2002/03 season [13].

Importantly the Australian−New Zealand H3N2 K viruses were still susceptible to all licensed influenza antiviral drugs from testing performed at the WHO Centre (71/71 viruses tested with oseltamvir, zanamivir, laninamivir, peramivir and 240/240 virus sequenced for baloxavir marboxil mutations; full data not shown). Hence, these antivirals may be used to ameliorate the outcomes from subclade K virus infections. These drugs are most effective if administered within 48 hours of when symptoms first appear [14] and may have an increased role in treating severe infections. Encouragingly, despite high apparent transmissibility, there is no evidence to date of a clinical severity signal with K viruses. Additionally, a preliminary UK study found the typical range of vaccine effectiveness (VE) in line with age groups (2−12 years, 18−64 years and ≥ 65 years) against emergency department attendance or hospitalisation, during the early part of the season (29 September−2 November 2025) when K viruses were 87% prevalent [15]. If these VEs are borne out, then influenza vaccination will still be useful in reducing the impact of the disease.

This analysis has limitations. Most influenza A samples in both Australia and New Zealand are not subtyped and only a small proportion of influenza A(H3N2) viruses have had isolates generated and tested in HI assays and similarly only a fraction of viruses was sequenced and analysed phylogenetically. Additionally, only international HA and NA influenza sequences that were available on GISAID at the time of analysis were included in this study.

## Conclusion

Given the speed and size of the outbreaks of K viruses in Australia−New Zealand and the near global spread of these viruses already, it is likely that they will further expand during the NH winter season and persist for the remainder of 2025 and into 2026. Careful clinical and epidemiological monitoring combined with timely virus sequencing and further VE studies, will determine the extent and impact that this new influenza A(H3N2) variant will have over the coming months, but countries should be prepared for increased demands on their healthcare systems if this variant predominates, as one might expect it will, based on current global trends.

## Data Availability

See Supplementary Material for a link to the full list of sequences used in this study.

## Supplementary Data

2151000 Supplementary Materials
