# Erratum for Euro Surveill. 2025;30(49)

**DOI:** 10.2807/1560-7917.ES.2025.30.50.251218c

**Published:** 2025-12-18

**Authors:** Eurosurveillance editorial team

**Affiliations:** 1 European Centre for Disease Prevention and Control (ECDC), Stockholm, Sweden

In the article entitled ‘Extended influenza seasons in Australia and New Zealand in 2025 due to the emergence of influenza A(H3N2) subclade K viruses’ by Dapat et al., published on 11 December 2025, the differences in the thicknesses (i.e. widths) of the blue lines in Figure 3, which should have been clear, were not visible, with all lines seemingly having the same width. The Figure 3 was corrected on 18 December 2025. We apologise for the mistake.

