# Supplementary material for: Extended influenza seasons in Australia and New Zealand in 2025 due to the emergence of influenza A(H3N2) subclade K viruses
Source: Euro Surveill. 2025 Dec 11;30(49):2500894. doi: 10.2807/1560-7917.ES.2025.30.49.2500894 (PMC12701333; doi:10.2807/1560-7917.ES.2025.30.49.2500894)
Supplement: Supplementary Materials [file Supplementary_Materials_2500894.pdf]

Supplementary Material

Extended influenza seasons in Australia and New Zealand in 2025 due to the emergence of influenza A(H3N2) subclade K viruses

This supplementary material is hosted by Eurosurveillance as supporting information alongside the article "Extended influenza seasons in Australia and New Zealand in 2025 due to the emergence of influenza A(H3N2) subclade K viruses", on behalf of the authors, who remain responsible for the accuracy and appropriateness of the content. The same standards for ethics, copyright, attributions and permissions as for the article apply. Supplements are not edited by Eurosurveillance and the journal is not responsible for the maintenance of any links or email addresses provided therein."

Sequence annotation

A total of 21,406 influenza A(H3N2) sequences collected between 01 January 2025 and 11 November 2025 were downloaded from GISAID (accessed 11 November 2025, see Supplementary file: GISAID Acknowledgment). Assignment of clades and subclades was performed using Nextclade v3.18.0 (Aksamentov, et al.).

Phylogenetic analysis (Supplementary Figure 1)

Sequences from Australia and New Zealand were aligned using MAFFT v7.526 (Nakamura et al.). Representative reference sequences from major subclades were included as a backbone for phylogenetic inference. Phylogenetic trees of the HA and NA genes were inferred using the maximum likelihood method as implemented in IQ-TREE2 v2.3.3 (Minh et al.). Time-scaled trees were estimated using the least-square dating method (To et al.). Branch support was assessed with 1,000 replicates using the ultrafast bootstrap method (Hoang et al.). Trees were visualised using the ggtree v3.10.1 package (Yu) in R v4.3.3.

The HA and NA phylogenies showed that subclade K (HA) and B.4.2.2 (NA) were the predominant viruses circulating in Australia and New Zealand in 2025 (Supplementary Figure S1).

Phylogeographic analysis (Figure 3)

Temporal signal was assessed using a root-to-tip regression analysis on subclade K viruses in TempEst v1.5.3 (Rambaut et al.). Result showed a correlation coefficient of 0.62 and an R^2 of 0.39, supporting sufficient a molecular clock signal for subsequent analysis (Supplementary Figure S3). Phylogeographic reconstruction was performed using BEAST X v10.0.0 (Baele et al.) with a discrete trait model to estimate migration rates between countries. BEAST analyses were run under an HKY+Γ nucleotide substitution model, an uncorrelated lognormal relaxed molecular clock, and a GMRF Bayesian Skyride tree prior. Migration rates were modelled as a symmetric discrete trait process with Bayesian stochastic search variable selection (Lemey et al.). Three independent MCMC chains of 500 million steps were run, sampling every 50,000 steps. Convergence and effective sample sizes (ESS > 200) were verified using Tracer v1.7.2 (Rambaut et al.). Results from all runs were combined using LogCombiner v1.8 following removal of the first 10% as burn-in. Bayes factors (Lemey et al.) were calculated using SPREAD v0.9.7 (Bielejec et al.) to identify migration pathways with strong statistical support. Migration routes with Bayes factors >3 and corresponding posterior probabilities were visualised on a Natural Earth world map (www.naturalearthdata.com) using the GeoPandas v1.1.1 package in Python v3.14.

The strongest supported migration event was between Australia and New Zealand (Bayes factor value = 108, Figure 3). A well-supported link was observed between Australia and the USA (Bayes factor = 25). These findings suggest that subclade K viruses may have emerged in the USA, subsequently spread to Australia and then to New Zealand. However, the analysis should be interpreted with caution due to potential sampling bias with sequences available at GISAID.

Ferret and Human serology

These assays were performed as previously described (Diefenbach-Elstob et al.). Briefly, the antigenic properties of influenza viral isolates were analysed using the haemagglutination inhibition (HI) assay. With this assay viruses were tested for their ability to agglutinate Guinea pig red blood cells (RBC) after incubation for 45 minutes with different post-infection ferret anti-sera raised against several reference viruses. HI assays A(H3N2) viruses were performed in the presence of 20 nM oseltamivir carboxylate to reduce non-specific binding by the NA protein. For human serology ferret antisera were replaced with sera obtained from subjects vaccinated with the 2025 Australian influenza vaccine which contained an A/Croatia/10136RV/2023-like virus. The human sera was kindly provided by CSL Seqirus Limited who conducted the study. Sera was collected 21-28 days after vaccination from adults (18-64y and 65y+). All sera were treated with receptor-destroying enzyme (RDE) to remove non-specific binding prior to performing the HI. Ferret and human sera were serially diluted starting at a dilution of 1:10 or more and the titre of the sera was determined to be the reciprocal of last dilution that gave full inhibition of RBC agglutination.

Next generation sequencing

Details on sequencing are as previously described (Diefenbach-Elstob et al.). For sequencing, RNA was extracted from isolates or original clinical specimens using either a manual QIAGEN QIAamp Viral RNA kit or the automated QIAGEN QIA Xtractor platform. Most samples were sequenced using the multi-fragment RT-PCR (mRT-PCR) for either WGS or targeted sequencing of the HA, NA, and PA genes for influenza A, or the HA, NA and PA genes for influenza B, using SuperScriptIV one-step RT-PCR System (ThermoFisher) with primer sets as described previ-ously. NGS was conducted using an Illumina iSeq100 or the Oxford Minion Flowcells according to the manufacturer’s recommendations. Sequence data was analysed using an adaption of the IRMA pipeline. Phylogenetic analysis was performed using the Augur pipeline,15 and trees were constructed using IQ-TREE 2,16 with 1,000 bootstrap replicates and generalized-time reversible (GTR) model and visualised using ggtree. Some viruses were sequenced externally with results provided by the submitting laboratory.

HA clade nomenclature was updated for A(H1N1)pdm09 and A(H3N2) viruses during 2023. Consequently, all clades were verified against data uploaded toNextclade and/or against internal or external sequencing results (for viruses not uploaded to Nextclade).

Epidemiology

Influenza notification data were obtained from the Australia Government Department of Health, Disability, and Ageing; and the New Zealand Institute for Public Health and Forensic Science. Demographic and clinical data were provided by laboratories submitting samples to the WHO Collaborating Centre for Reference and Research on Influenza. Influenza notifications were plotted over time for the period from 2022 to 2025. Pearson’s Chi-squared test was used to test for associations between demographic and clinical factors and influenza A(H3N2) subclades. Epidemiological plots were generated and statistical analyses were undertaken using R.

References

Aksamentov I, Roemer C, Hodcroft EB, and Neher RA. Nextclade: clade assignment, mutation calling and quality control for viral genomes. Journal of Open Source Software 2021, 6(67), 3773.

Baele G, Ji X, Hassler GW, McCrone JT, Shao Y, Zhang Z, Holbrook AJ, Lemey P, Drummond AJ, Rambaut A, Suchard MA. BEAST X for Bayesian phylogenetic, phylogeographic and phylodynamic inference. Nat Methods. 2025 Aug;22(8):1653-1656.

Bielejec F, Rambaut A, Suchard MA, Lemey P. SPREAD: spatial phylogenetic reconstruction of evolutionary dynamics. Bioinformatics. 2011 Oct 15;27(20):2910-2.

Diefenbach-Elstob TR, Lay O, Zakis T, Deshpande N, Soppe S, Peck H, et al. Report on influenza viruses received and tested by the Melbourne WHO Collaborating Centre for Reference and Research on Influenza during 2023. Commun Dis Intell (2018). 2025 May 19;49.

Hoang, DT, Chernomor O, Haeseler A, Minh BQ, Vinh LS. UFBoot2: Improving the Ultrafast Bootstrap Approximation. Mol Biol Evol. 2018 Feb 1;35(2):518-522.

Lemey P, Rambaut A, Drummond AJ, Suchard MA. Bayesian phylogeography finds its roots. PLoS Comput Biol. 2009 Sep;5(9):e1000520.

Minh BQ, Schmidt HA, Chernomor O, Schrempf D, Woodhams MD, von Haeseler A, Lanfear R. IQ-TREE 2: New Models and Efficient Methods for Phylogenetic Inference in the Genomic Era. Mol Biol Evol. 2020 May 1;37(5):1530-1534

Nakamura T, Yamada KD, Tomii K, Katoh K.Parallelization of MAFFT for large-scale multiple sequence alignments. Bioinformatics. 2018 Jul 15;34(14):2490-2492

Rambaut A, Lam TT, Carvalho LM, Pybus OG. Exploring the temporal structure of heterochronous sequences using TempEst (formerly Path-O-Gen). Virus Evol. 2016 Apr 9;2(1):vew007.

Rambaut A, Drummond AJ, Xie D, Baele G, Suchard MA. Posterior Summarization in Bayesian Phylogenetics Using Tracer 1.7. Syst Biol. 2018 Sep 1;67(5):901-904.

To T, Jung M, Lycett S, Gascuel O, Fast Dating Using Least-Squares Criteria and Algorithms. Syst Biol. 2016 Jan;65(1):82-97.

Yu G. Using ggtree to Visualize Data on Tree-Like Structures. Curr Protoc Bioinformatics. 2020 Mar;69(1):e96.

Supplementary Figures and Tables follow.

Supplementary Figure 1.
HA and NA H3 phylogenetic trees with emphasis on 2025 viruses.

Supplementary Figure 2
HI geometric mean titres from post-vaccination subjects who received the Australian 2025 influenza vaccine (Adult cell (n=25) or egg based standard vaccine (n=25) or for Elderly egg based vaccine with the MF-59 adjuvanted vaccine (n=20). These sera were selected for good reactivity with the 2025 A(H3N2) vaccine virus and may not be representative of the total population. Names on the top and bottom of the panels represent the virus designation and the medium in which the viruses were propagated eg E3= egg passage 3, QMC2=Qualified manufacturing cells, passage 2. The H3N2 subclade is also marked eg J.2, J.2.1 or J.2.4 or K. The dotted line represents a 50% reduction in titre with respect to the cell grown vaccine component A/District of Columbia/27/2023 which is an A/Croatia/10136RV/2023-like virus. Titres below 50% are considered to be arbitrarily significant. Bars shown in green are above the 50% cut off and those in navy blue fall below the

50% cut off. VIDRL stands for Victorian Infectious Diseases Reference Laboratory, the host for the WHO influenza Centre, and simply signifies the panels used.

Supplementary Figure 3. Root-to-regression analysis of influenza A(H3N2) subclade K viruses against sample collection date.

Supplementary Table 1.
A representative table of the haemagglutination inhibition titres of ferret post-infection antisera raised against various subclades of influenza A(H3N2) reacting with 2025 New Zealand virus isolates. See Table 1 for abbreviations.

Supplementary Table 2
Demographics of Australian subjects infected with H3N2 by subclade if it was known.

Supplementary Table 3.
Hospitalisations with influenza A(H3N2) at Westmead Children's Hospital, NSW, Australia: 1 January to 31 October 2025.

A. Influenza A(H3N2) HA Supplementary figure 1

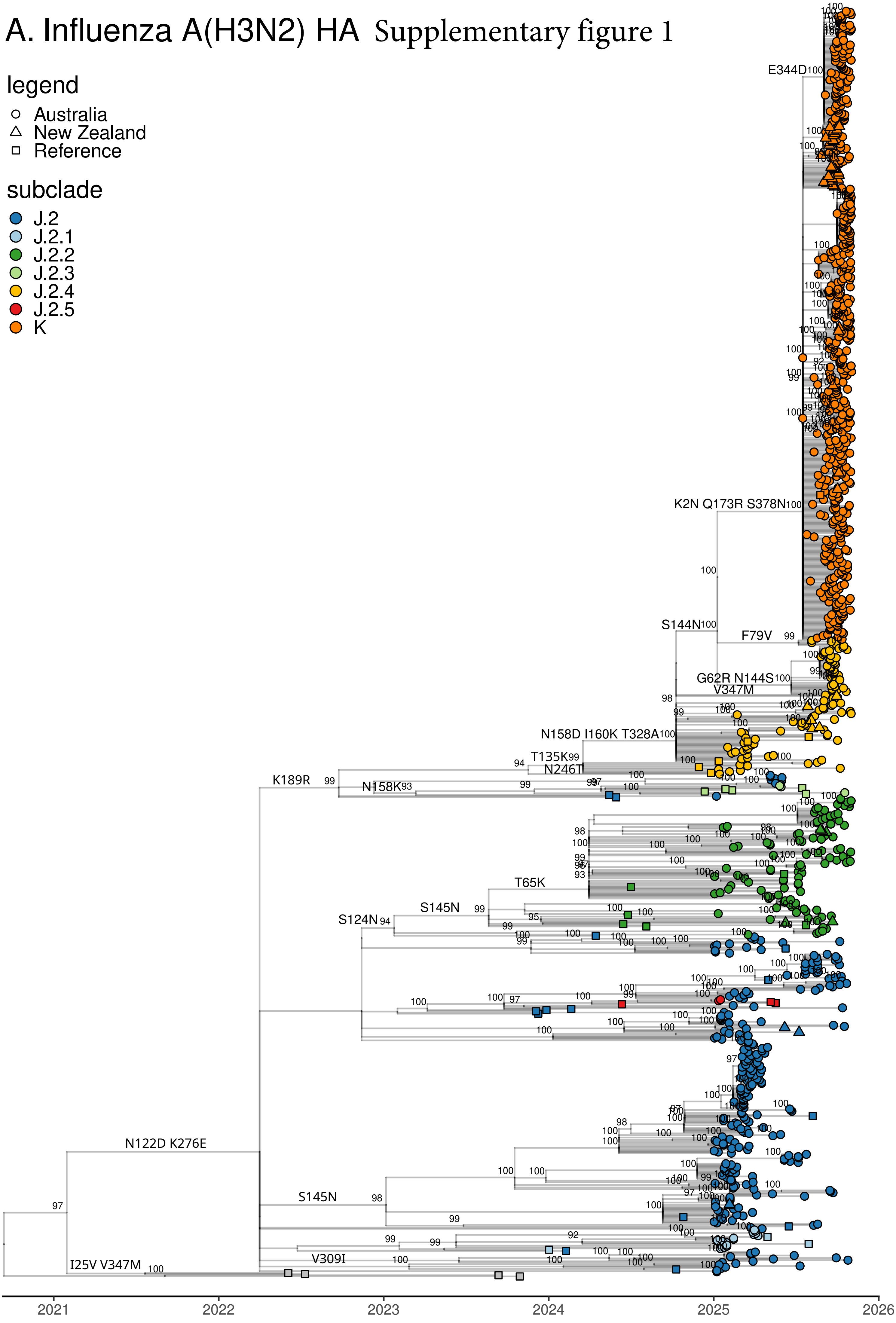

B. Influenza A(H3N2) NA Supplementary figure 1

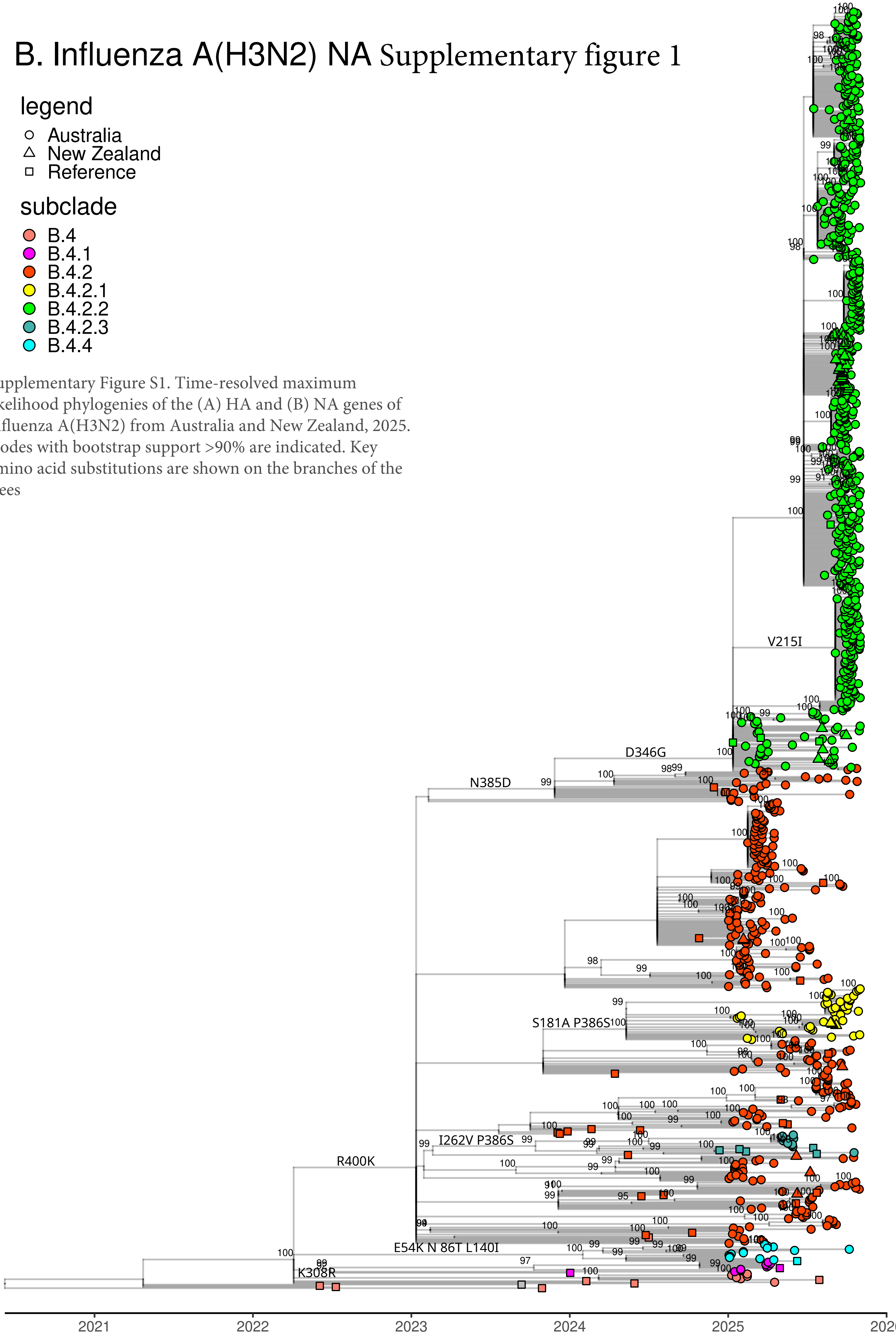

Supplementary Figure S1. Time-resolved maximum likelihood phylogenies of the (A) HA and (B) NA genes of influenza A(H3N2) from Australia and New Zealand, 2025. Nodes with bootstrap support >90% are indicated. Key amino acid substitutions are shown on the branches of the trees

Compared with A/District of Columbia/27/2023 cell

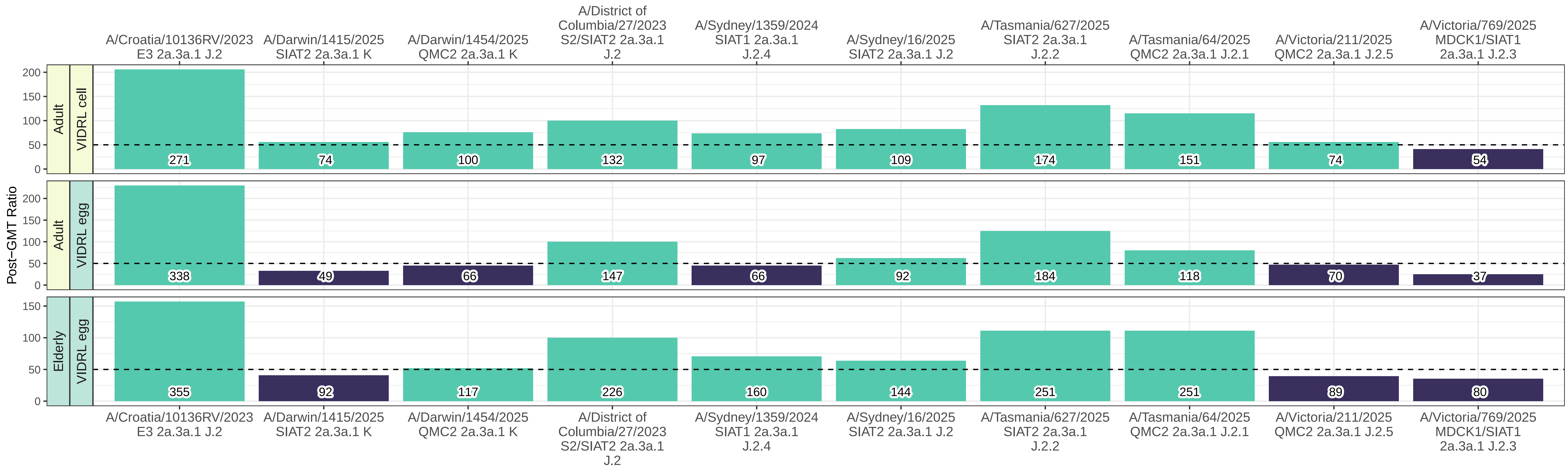

Supplementary Figure 3

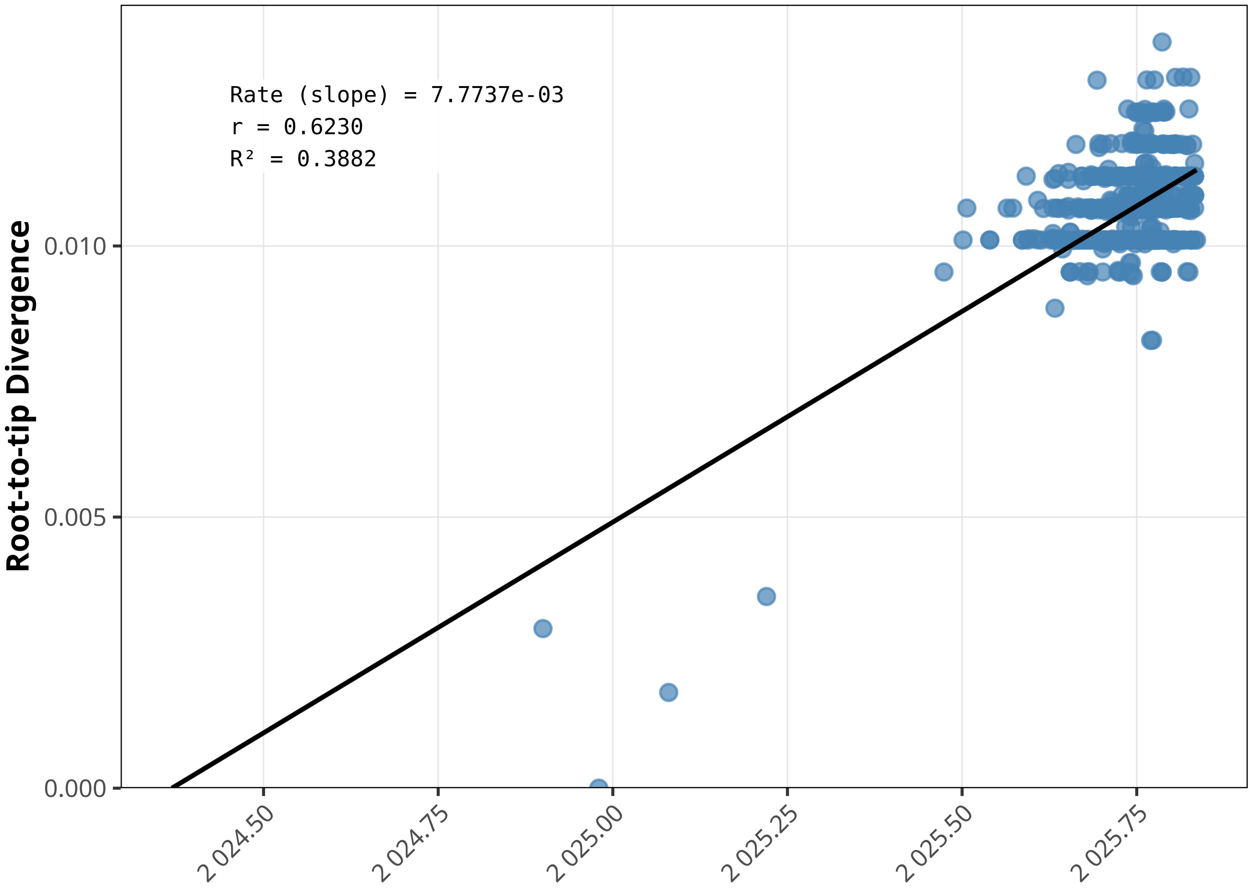

Supplementary Figure S3. Root-to-regression analysis of influenza A(H3N2) subclade K viruses against sample collection date.

H3N2, HI
07/11/2025

Supplementary Table 1

Fold difference
- <4−fold
- 4−fold
- 8−fold
- >8−fold

	SIAT3,SIAT4	E3	E4	SIAT1	MDCK1			
	Cro10136RV	Cro10136RV	SingGP20238	Syd1359	Darwin1415			
	A9936	A9933	A10145	A9985	A10162		Passage	Sample
	J.2	J.2	J.2.4	J.2.4	K	Subclade	details	Date
REFERENCE ANTIGENS								
A/Croatia/10136RV/2023 c	**320**	320	80	40	40	J.2	SIAT3,SIAT6	
A/Croatia/10136RV/2023e	1280	**2560**	320	160	40	J.2	E3	
A/Singapore/GP20238/2024e	160	320	**2560**	640	640	J.2.4	E5	
A/Sydney/1359/2024c	160	320	2560	**640**	640	J.2.4	SIAT1	
A/Darwin/1415/2025c	40	40	1280	320	**1280**	K	SIAT2	
TEST ANTIGENS								
A/Auckland/106/2025	320	640	80	40	<40	J.2	SIATX,SIAT1	2025−07−09
A/Nelson/02/2025	320	640	80	40	<40	J.2	SIAT1	2025−06−07
A/Auckland/101/2025	320	640	80	40	<40	J.2.2	SIAT1	2025−09−21
A/Canterbury/13/2025	320	640	80	40	40	J.2.2	SIAT1	2025−09−07
A/Canterbury/20/2025	320	640	40	40	<40	J.2.2	SIATX,SIAT1	2025−06−09
A/Hawkes Bay/07/2025	320	640	80	80	80	J.2.2	SIATX,SIAT1	2025−08−25
A/Hawkes Bay/08/2025	320	640	80	40	40	J.2.2	SIATX,SIAT1	2025−08−29
A/Wairarapa/02/2025	80	160	2560	320	640	J.2.4	SIAT1	2025−09−30
A/Wairarapa/01/2025	40	160	2560	640	320	J.2.4	SIAT1	2025−07−28
A/Wellington/44/2025	<40	80	640	320	1280	K	SIAT1	2025−09−23
A/Auckland/103/2025	<40	40	1280	320	1280	K	SIAT1	2025−10−04
A/Auckland/104/2025	<40	40	1280	320	1280	K	SIAT1	2025−10−01
A/Bay of Plenty/03/2025	<40	40	1280	160	640	K	SIAT1	2025−09−02
A/Canterbury/19/2025	<40	40	640	160	640	K	SIAT1	2025−10−04
A/Dunedin/08/2025	<40	40	640	160	640	K	SIAT1	2025−09−26
A/Dunedin/10/2025	<40	40	1280	320	1280	K	SIAT1	2025−10−05
A/Nelson/08/2025	<40	40	640	160	640	K	SIAT1	2025−10−02
A/Taranaki/02/2025	<40	40	1280	320	1280	K	SIAT1	2025−09−22
A/Waikato/01/2025	<40	40	1280	320	1280	K	SIAT1	2025−09−15

Supplementary Table 2

	A(H3N2) subclades								
	**Subclade not known**	**J.2**	**J.2.1**	**J.2.2**	**J.2.3**	**J.2.4**	**J.2.5**	**K**	**p-test***
n	421	261	15	107	4	107	2	502	
**Sex (%)**									
Female	210 (49.9)	131 (50.2)	7 (46.7)	42 (39.3)	1 (25.0)	42 (39.3)	1 (50.0)	276 (55.0)	<0.001
Male	210 (49.9)	118 (45.2)	5 (33.3)	59 (55.1)	2 (50.0)	63 (58.9)	1 (50.0)	226 (45.0)	
NA	1 (0.2)	12 (4.6)	3 (20.0)	6 (5.6)	1 (25.0)	2 (1.9)	0 (0.0)	0 (0.0)	
**Age group (%)**									
0-<5	114 (27.1)	55 (21.1)	4 (26.7)	27 (25.2)	0 (0.0)	24 (22.4)	1 (50.0)	81 (16.1)	<0.001
5-<15	125 (29.7)	42 (16.1)	0 (0.0)	12 (11.2)	0 (0.0)	19 (17.8)	0 (0.0)	129 (25.7)	
15-<35	68 (16.2)	54 (20.7)	0 (0.0)	11 (10.3)	0 (0.0)	15 (14.0)	0 (0.0)	103 (20.5)	
35-<65	54 (12.8)	53 (20.3)	2 (13.3)	23 (21.5)	0 (0.0)	21 (19.6)	1 (50.0)	86 (17.1)	
>=65	60 (14.3)	45 (17.2)	6 (40.0)	27 (25.2)	3 (75.0)	22 (20.6)	0 (0.0)	86 (17.1)	
NA	0 (0.0)	12 (4.6)	3 (20.0)	7 (6.5)	1 (25.0)	6 (5.6)	0 (0.0)	17 (3.4)	
**Sample month (%)**									
Jan	35 (8.3)	47 (18.0)	7 (46.7)	8 (7.5)	0 (0.0)	2 (1.9)	2 (100.0)	0 (0.0)	<0.001
Feb	59 (14.0)	44 (16.9)	2 (13.3)	5 (4.7)	0 (0.0)	7 (6.5)	0 (0.0)	0 (0.0)	
Mar	12 (2.9)	74 (28.4)	2 (13.3)	3 (2.8)	0 (0.0)	15 (14.0)	0 (0.0)	0 (0.0)	
Apr	10 (2.4)	26 (10.0)	4 (26.7)	2 (1.9)	0 (0.0)	1 (0.9)	0 (0.0)	0 (0.0)	
May	7 (1.7)	12 (4.6)	0 (0.0)	11 (10.3)	2 (50.0)	4 (3.7)	0 (0.0)	0 (0.0)	
Jun	4 (1.0)	11 (4.2)	0 (0.0)	8 (7.5)	0 (0.0)	1 (0.9)	0 (0.0)	0 (0.0)	
Jul	8 (1.9)	11 (4.2)	0 (0.0)	20 (18.7)	0 (0.0)	5 (4.7)	0 (0.0)	3 (0.6)	
Aug	31 (7.4)	13 (5.0)	0 (0.0)	18 (16.8)	0 (0.0)	11 (10.3)	0 (0.0)	16 (3.2)	
Sep	69 (16.4)	12 (4.6)	0 (0.0)	13 (12.1)	0 (0.0)	45 (42.1)	0 (0.0)	159 (31.7)	
Oct	127 (30.2)	11 (4.2)	0 (0.0)	17 (15.9)	1 (25.0)	15 (14.0)	0 (0.0)	314 (62.5)	
Nov	59 (14.0)	0 (0.0)	0 (0.0)	1 (0.9)	0 (0.0)	1 (0.9)	0 (0.0)	10 (2.0)	
NA	0 (0.0)	0 (0.0)	0 (0.0)	1 (0.9)	1 (25.0)	0 (0.0)	0 (0.0)	0 (0.0)	
**Sender state (%)**									
ACT	15 (3.6)	15 (5.7)	0 (0.0)	1 (0.9)	0 (0.0)	0 (0.0)	0 (0.0)	11 (2.2)	<0.001
NSW	108 (25.7)	19 (7.3)	1 (6.7)	8 (7.5)	0 (0.0)	9 (8.4)	0 (0.0)	38 (7.6)	
NT	79 (18.8)	75 (28.7)	1 (6.7)	20 (18.7)	0 (0.0)	11 (10.3)	0 (0.0)	83 (16.5)	
QLD	25 (5.9)	24 (9.2)	7 (46.7)	10 (9.3)	0 (0.0)	4 (3.7)	0 (0.0)	4 (0.8)	

SA	16 (3.8)	70 (26.8)	4 (26.7)	40 (37.4)	1 (25.0)	46 (43.0)	0 (0.0)	308 (61.4)	
TAS	26 (6.2)	12 (4.6)	1 (6.7)	15 (14.0)	0 (0.0)	4 (3.7)	0 (0.0)	17 (3.4)	
VIC	145 (34.4)	41 (15.7)	1 (6.7)	10 (9.3)	2 (50.0)	24 (22.4)	2 (100.0)	32 (6.4)	
WA	7 (1.7)	4 (1.5)	0 (0.0)	3 (2.8)	0 (0.0)	7 (6.5)	0 (0.0)	9 (1.8)	
NA	0 (0.0)	1 (0.4)	0 (0.0)	0 (0.0)	1 (25.0)	2 (1.9)	0 (0.0)	0 (0.0)	
**Setting (%)**									
Community GP	18 (4.3)	13 (5.0)	1 (6.7)	8 (7.5)	0 (0.0)	9 (8.4)	0 (0.0)	20 (4.0)	<0.001
Emergency	178 (42.3)	98 (37.5)	2 (13.3)	33 (30.8)	0 (0.0)	22 (20.6)	1 (50.0)	107 (21.3)	
Hospital Outpatient	8 (1.9)	4 (1.5)	0 (0.0)	3 (2.8)	0 (0.0)	0 (0.0)	0 (0.0)	0 (0.0)	
Hospitalised Inpatient	205 (48.7)	63 (24.1)	5 (33.3)	14 (13.1)	2 (50.0)	31 (29.0)	1 (50.0)	76 (15.1)	
Residential Facility	0 (0.0)	1 (0.4)	1 (6.7)	0 (0.0)	0 (0.0)	0 (0.0)	0 (0.0)	0 (0.0)	
NA	12 (2.9)	82 (31.4)	6 (40.0)	49 (45.8)	2 (50.0)	45 (42.1)	0 (0.0)	299 (59.6)	

*Pearson’s chi-squared test
**Medical risk factors coded as 'yes' if the received sample indicated that the patients was immunocompromised, pregnant or had cardiac disease, obesity, diabetes or chronic kidney disease.

Supplementary Table 3.

Hospitalisations** with influenza A(H3N2) at Westmead Children's Hospital, NSW, Australia: 1 January to 31 October 2025 (n=71)

Subclade/ subtype	Jan	Feb	Mar	Apr	May	Jun	Jul	Aug	Sep	Oct	Total
H3 K	0	0	0	0	0	0	0	0	11	28	39
H3 non-K	3	6	3	0	1	0	5	3	5	4 (	30
Total H3 cases	3	6	3	0	1	0	5	3	17**	33**	71**

*Only 1 ICU case with H3N2 occurred in March 2025 with a non-K virus.

** Note: two cases recorded with unknown H3N2 subtype (September, n=1; October, n=1).

Data Availability

GISAID Identifier: EPI_SET_251124sn
DOI: https://doi.org/10.55876/gis8.251124sn

All genome sequences and associated metadata in this dataset are published in GISAID's EpiFlu database. To view the contributors of each individual sequence with details such as accession number, Virus name, Collection date, Originating Lab and Submitting Lab and the list of Authors, visit EPI_SET_251124sn

Data Snapshot

EPI_SET_251124sn is composed of 21406 individual viruses.
The collection dates range from 2025-01-01 to 2025-11-02;
Data were collected in 113 countries and territories.
